# (2*E*)-1-(6-Chloro-2-methyl-4-phenyl­quinolin-3-yl)-3-(4-chloro­phen­yl)prop-2-en-1-one

**DOI:** 10.1107/S1600536811004740

**Published:** 2011-02-12

**Authors:** S. Sarveswari, V. Vijayakumar, Seik Weng Ng, Edward R. T. Tiekink

**Affiliations:** a Organic Chemistry Division, School of Advanced Sciences, VIT University, Vellore 632 014, Tamilnadu, India; b Department of Chemistry, University of Malaya, 50603 Kuala Lumpur, Malaysia

## Abstract

Two independent mol­ecules comprise the asymmetric unit of the title chalcone, C_25_H_17_Cl_2_NO, and while each has an *E* configuration about the ethyl­ene double bond, they differ in the relative orientations of the carbonyl and ethyl­ene double bonds within the prop-2-en-1-one residues, *i.e. anti* and *syn*. For each mol­ecule, the benzene [dihedral angles = 71.04 (9) and 73.34 (12)°] and prop-2-en-1-one [C—C—C—O = 91.2 (2) and −119.1 (3)°] substituents are twisted out of the plane of the quinoline moiety to which they are attached. The crystal structure is stabilized by C—H⋯π and π–π [*Cg*(quinoline)⋯*Cg*(quinoline) = 3.7809 (12) and 3.8446 (11) Å] inter­actions.

## Related literature

For background to chalcone chemistry, see: Roman (2004 ▶). For related structures, see: Prasath *et al.* (2010 ▶); Reddy *et al.* (2010 ▶). 
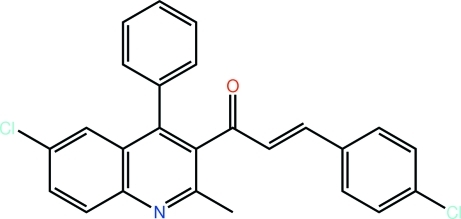


## Experimental

### 

#### Crystal data

C_25_H_17_Cl_2_NO
*M* *_r_* = 418.30
Triclinic, 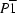

*a* = 11.1704 (3) Å
*b* = 12.8497 (5) Å
*c* = 16.0591 (6) Å
α = 74.914 (3)°
β = 80.603 (3)°
γ = 70.789 (3)°
*V* = 2094.05 (13) Å^3^
*Z* = 4
Cu *K*α radiation
μ = 2.91 mm^−1^
*T* = 295 K
0.30 × 0.30 × 0.10 mm

#### Data collection

Agilent Supernova Dual diffractometer with an Atlas detector
Absorption correction: multi-scan (*CrysAlis PRO*; Agilent, 2010 ▶) *T*_min_ = 0.574, *T*_max_ = 1.000
14958 measured reflections
8252 independent reflections
7088 reflections with *I* > 2σ(*I*)
*R*_int_ = 0.026

#### Refinement

*R*[*F* ^2^ > 2σ(*F* ^2^)] = 0.052
*wR*(*F* ^2^) = 0.158
*S* = 1.03
8252 reflections
525 parameters
H-atom parameters constrained
Δρ_max_ = 0.43 e Å^−3^
Δρ_min_ = −0.41 e Å^−3^

### 

Data collection: *CrysAlis PRO* (Agilent, 2010 ▶); cell refinement: *CrysAlis PRO*; data reduction: *CrysAlis PRO*; program(s) used to solve structure: *SHELXS97* (Sheldrick, 2008 ▶); program(s) used to refine structure: *SHELXL97* (Sheldrick, 2008 ▶); molecular graphics: *ORTEP-3* (Farrugia, 1997 ▶) and *DIAMOND* (Brandenburg, 2006 ▶); software used to prepare material for publication: *publCIF* (Westrip, 2010 ▶).

## Supplementary Material

Crystal structure: contains datablocks global, I. DOI: 10.1107/S1600536811004740/hg2798sup1.cif

Structure factors: contains datablocks I. DOI: 10.1107/S1600536811004740/hg2798Isup2.hkl

Additional supplementary materials:  crystallographic information; 3D view; checkCIF report

## Figures and Tables

**Table 1:** Hydrogen-bond geometry (Å, °) *Cg*1 is the centroid of the C20–C25 ring.

*D*—H⋯*A*	*D*—H	H⋯*A*	*D*⋯*A*	*D*—H⋯*A*
C38—H38⋯*Cg*1^i^	0.93	2.93	3.458 (3)	117

Symmetry code: (i) 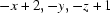.

**Fig. 1.**
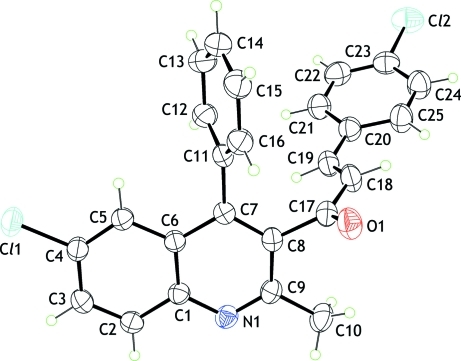
The molecular structure of the first independent molecule of (I), i.e. the anti form, showing the atom-labelling scheme and displacement ellipsoids at the 35% probability level.

**Fig. 2.**
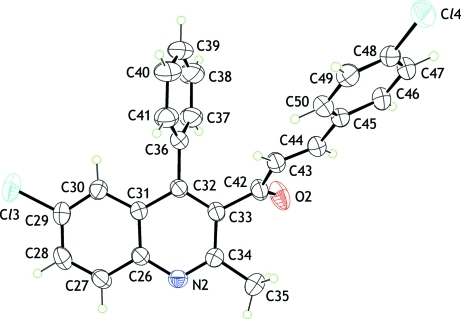
The molecular structure of the second independent molecule of (I), i.e. the syn form, showing the atom-labelling scheme and displacement ellipsoids at the 35% probability level.

**Fig. 3.**
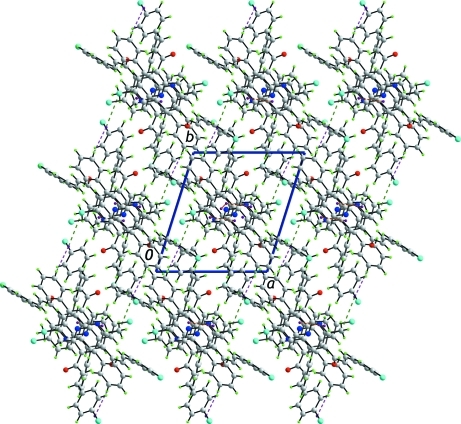
A view in projection down the c axis of the unit-cell contents of (I). The C—H···π, π–π, and C—Cl···π interactions are shown as purple, orange and green dashed lines, respectively.

## supplementary crystallographic information

### Comment

Chalcones and its analogs are valuable intermediates in organic synthesis and exhibit a multitude of biological activities. From a chemical point of view, an important feature of chalcones and their heteroanalogs is the ability to act as activated unsaturated systems in conjugated addition reactions of carbanions in the presence of basic catalysts (Roman, 2004). The title compound, (I), was examined in continuation of our interest in the structural chemistry of chalcones (Prasath *et al.*, 2010; Reddy *et al.*, 2010).

Two independent molecules comprise the asymmetric unit of (I), one with an *anti* relationship between the carbonyl and ethylene double bonds, Fig. 1, and one with a *syn* relationship, Fig. 2. In each, the conformation about the ethylene bond [C18═C19 = 1.319 (3) Å and C43═C44 = 1.328 (3) Å] is *E*. For both molecules, the benzene ring is twisted out of the plane of the quinoline residue to which it is connected; the C6—C7—C11—C12 and C31—C32—C36—C37 torsion angles are -69.0 (2) and -107.1 (2) °, respectively. The prop-2-en-1-one substituents are also twisted out of the plane of the respective quinoline residues as seen in the values of the C7—C8—C17—O1 and C32—C33—C42—O2 torsion angles of 91.2 (2) and -119.1 (3) °, respectively. Within the prop-2-en-1-one substituents themselves, the terminal benzene rings are not co-planar with the C18—C19—C20—C21 and C43—C44—C45—C46 torsion angles being -159.6 (2) and -170.8 (2) °, respectively.

The molecules are stabilized in the crystal packing by a combination of C—H···π, π–π, and C—Cl···π interactions. The C—H···π contacts, Table 1, occur between the two molecules comprising the asymmetric unit. The π–π interactions occur between centrosymmetrically related quinoline rings belonging to like molecules [*Cg*(N1,C1,C6—C9)···*Cg*(C1—C6)^ii^ = 3.8446 (11) ° for *ii*: 1 - *x*, 1 - *y*, -*z*; and *Cg*(N2,C26,C31—C34)···*Cg*(C26—C31)^iii^ = 3.7809 (12) ° for *iii*: 1 - *x*, 1 - *y*, 1 - *z*]. The C—H···Cl contacts [C4—Cl1···*Cg*(C20—C25)^iv^ = 3.6082 (13) Å and angle at Cl1 = 116.34 (7) ° for *iv*: -1 + *x*, *y*, *z*] also occur between like molecules. A view of the crystal packing is shown in Fig. 3.

### Experimental

A mixture of 3-acetyl-6-chloro-2-methyl-4-phenylquinoline (3.1 g, 0.01 *M*) and 4-chlorobenzaldehyde (1.4 g, 0.01 *M*) and a catalytic amount of KOH in distilled ethanol (40 ml) was stirred for about 12 h. The resulting mixture was concentrated to remove ethanol, poured onto ice and neutralized with dilute acetic acid. The resultant solid was filtered, dried and purified by column chromatography using a 1:1 mixture of ethyl acetate and petroleum ether. Recrystallization was from acetone; Yield: 64% and m.pt: 397–399 K.

### Refinement

Carbon-bound H-atoms were placed in calculated positions (C—H 0.93 to 0.96 Å) and were included in the refinement in the riding model approximation, with *U*_iso_(H) set to 1.2 to 1.5*U*_equiv_(C).

### Crystal data

**Table:**

C_25_H_17_Cl_2_NO	*Z* = 4
*M**_r_* = 418.30	*F*(000) = 864
Triclinic, *P*1	*D*_x_ = 1.327 Mg m^−^^3^
Hall symbol: -P 1	Cu *K*α radiation, λ = 1.54184 Å
*a* = 11.1704 (3) Å	Cell parameters from 8901 reflections
*b* = 12.8497 (5) Å	θ = 2.9–74.1°
*c* = 16.0591 (6) Å	µ = 2.91 mm^−^^1^
α = 74.914 (3)°	*T* = 295 K
β = 80.603 (3)°	Prism, colourless
γ = 70.789 (3)°	0.30 × 0.30 × 0.10 mm
*V* = 2094.05 (13) Å^3^	

### Data collection

**Table:**

Agilent Supernova Dual diffractometer with an Atlas detector	8252 independent reflections
Radiation source: SuperNova (Cu) X-ray Source	7088 reflections with *I* > 2σ(*I*)
Mirror	*R*_int_ = 0.026
Detector resolution: 10.4041 pixels mm^-1^	θ_max_ = 74.3°, θ_min_ = 2.9°
ω scans	*h* = −11→13
Absorption correction: multi-scan (*CrysAlis PRO*; Agilent, 2010)	*k* = −16→15
*T*_min_ = 0.574, *T*_max_ = 1.000	*l* = −20→19
14958 measured reflections	

### Refinement

**Table:**

Refinement on *F*^2^	Primary atom site location: structure-invariant direct methods
Least-squares matrix: full	Secondary atom site location: difference Fourier map
*R*[*F*^2^ > 2σ(*F*^2^)] = 0.052	Hydrogen site location: inferred from neighbouring sites
*wR*(*F*^2^) = 0.158	H-atom parameters constrained
*S* = 1.03	*w* = 1/[σ^2^(*F*_o_^2^) + (0.0899*P*)^2^ + 0.4585*P*] where *P* = (*F*_o_^2^ + 2*F*_c_^2^)/3
8252 reflections	(Δ/σ)_max_ = 0.001
525 parameters	Δρ_max_ = 0.43 e Å^−^^3^
0 restraints	Δρ_min_ = −0.41 e Å^−^^3^

### Special details

**Table:**

Geometry. All s.u.'s (except the s.u. in the dihedral angle between two l.s. planes) are estimated using the full covariance matrix. The cell s.u.'s are taken into account individually in the estimation of s.u.'s in distances, angles and torsion angles; correlations between s.u.'s in cell parameters are only used when they are defined by crystal symmetry. An approximate (isotropic) treatment of cell s.u.'s is used for estimating s.u.'s involving l.s. planes.
Refinement. Refinement of *F*^2^ against ALL reflections. The weighted *R*-factor *wR* and goodness of fit *S* are based on *F*^2^, conventional *R*-factors *R* are based on *F*, with *F* set to zero for negative *F*^2^. The threshold expression of *F*^2^ > 2σ(*F*^2^) is used only for calculating *R*-factors(gt) *etc*. and is not relevant to the choice of reflections for refinement. *R*-factors based on *F*^2^ are statistically about twice as large as those based on *F*, and *R*- factors based on ALL data will be even larger.

### Fractional atomic coordinates and isotropic or equivalent isotropic displacement parameters (Å^2^)

**Table:**

	*x*	*y*	*z*	*U*_iso_*/*U*_eq_	
Cl1	0.16358 (5)	0.49560 (5)	0.26030 (4)	0.06920 (17)	
Cl2	1.33991 (8)	0.11218 (7)	0.45986 (5)	0.0924 (2)	
O1	0.86575 (14)	0.22234 (15)	0.02125 (11)	0.0691 (4)	
N1	0.61911 (15)	0.56418 (13)	0.04091 (10)	0.0501 (4)	
C1	0.51604 (16)	0.54394 (15)	0.09308 (11)	0.0443 (4)	
C2	0.40157 (18)	0.63350 (16)	0.09149 (13)	0.0512 (4)	
H2	0.3986	0.7037	0.0554	0.061*	
C3	0.29485 (18)	0.61886 (17)	0.14218 (14)	0.0541 (4)	
H3	0.2196	0.6783	0.1404	0.065*	
C4	0.30038 (17)	0.51310 (16)	0.19691 (13)	0.0501 (4)	
C5	0.40840 (17)	0.42366 (16)	0.20038 (12)	0.0486 (4)	
H5	0.4092	0.3542	0.2370	0.058*	
C6	0.51918 (16)	0.43729 (14)	0.14790 (11)	0.0433 (4)	
C7	0.63491 (17)	0.34658 (15)	0.14543 (12)	0.0463 (4)	
C8	0.73523 (17)	0.36816 (16)	0.08963 (12)	0.0486 (4)	
C9	0.72457 (17)	0.47965 (17)	0.03906 (13)	0.0503 (4)	
C10	0.8367 (2)	0.5062 (2)	−0.01747 (17)	0.0686 (6)	
H10A	0.8075	0.5746	−0.0603	0.103*	
H10B	0.8815	0.4452	−0.0456	0.103*	
H10C	0.8926	0.5159	0.0174	0.103*	
C11	0.64371 (16)	0.23274 (15)	0.20244 (13)	0.0485 (4)	
C12	0.6432 (2)	0.21729 (18)	0.29087 (14)	0.0595 (5)	
H12	0.6381	0.2775	0.3146	0.071*	
C13	0.6502 (2)	0.11177 (19)	0.34435 (16)	0.0692 (6)	
H13	0.6508	0.1012	0.4038	0.083*	
C14	0.6564 (2)	0.02285 (19)	0.30963 (16)	0.0675 (6)	
H14	0.6597	−0.0474	0.3458	0.081*	
C15	0.6575 (2)	0.03717 (19)	0.22225 (17)	0.0665 (6)	
H15	0.6624	−0.0234	0.1990	0.080*	
C16	0.6514 (2)	0.14203 (18)	0.16806 (15)	0.0584 (5)	
H16	0.6524	0.1515	0.1086	0.070*	
C17	0.85560 (18)	0.27261 (18)	0.07789 (13)	0.0542 (4)	
C18	0.95710 (19)	0.2412 (2)	0.13521 (15)	0.0622 (5)	
H18	1.0323	0.1867	0.1230	0.075*	
C19	0.95068 (19)	0.28387 (19)	0.20273 (14)	0.0601 (5)	
H19	0.8771	0.3412	0.2127	0.072*	
C20	1.0485 (2)	0.24950 (19)	0.26344 (14)	0.0597 (5)	
C21	1.0179 (2)	0.2730 (2)	0.34513 (15)	0.0667 (6)	
H21	0.9357	0.3158	0.3598	0.080*	
C22	1.1072 (3)	0.2339 (2)	0.40532 (16)	0.0718 (6)	
H22	1.0853	0.2496	0.4601	0.086*	
C23	1.2288 (2)	0.1717 (2)	0.38295 (15)	0.0657 (5)	
C24	1.2642 (2)	0.1516 (3)	0.30134 (17)	0.0769 (7)	
H24	1.3477	0.1123	0.2862	0.092*	
C25	1.1743 (2)	0.1906 (2)	0.24211 (16)	0.0747 (7)	
H25	1.1981	0.1772	0.1867	0.090*	
Cl3	0.90535 (6)	0.58126 (7)	0.40484 (4)	0.0849 (2)	
Cl4	0.90415 (7)	−0.25246 (7)	1.15298 (5)	0.0979 (3)	
O2	0.4659 (2)	0.17598 (16)	0.71587 (14)	0.0901 (6)	
N2	0.45355 (15)	0.52142 (13)	0.63374 (11)	0.0513 (4)	
C26	0.56090 (18)	0.53029 (15)	0.58190 (12)	0.0473 (4)	
C27	0.5587 (2)	0.63632 (17)	0.52686 (14)	0.0595 (5)	
H27	0.4852	0.6972	0.5274	0.071*	
C28	0.6626 (2)	0.65091 (18)	0.47297 (14)	0.0641 (5)	
H28	0.6597	0.7208	0.4365	0.077*	
C29	0.7733 (2)	0.55961 (19)	0.47328 (13)	0.0578 (5)	
C30	0.78037 (19)	0.45574 (18)	0.52438 (13)	0.0530 (4)	
H30	0.8551	0.3963	0.5228	0.064*	
C31	0.67341 (17)	0.43863 (15)	0.57993 (11)	0.0452 (4)	
C32	0.67093 (17)	0.33154 (15)	0.63337 (12)	0.0474 (4)	
C33	0.55979 (18)	0.32275 (16)	0.68166 (12)	0.0488 (4)	
C34	0.45213 (18)	0.42134 (17)	0.68166 (12)	0.0500 (4)	
C35	0.3321 (2)	0.4172 (2)	0.73827 (16)	0.0666 (6)	
H35A	0.2747	0.4924	0.7340	0.100*	
H35B	0.3519	0.3848	0.7972	0.100*	
H35C	0.2929	0.3717	0.7198	0.100*	
C36	0.78570 (19)	0.23100 (16)	0.63245 (14)	0.0545 (4)	
C37	0.7881 (2)	0.1469 (2)	0.5930 (2)	0.0778 (7)	
H37	0.7175	0.1524	0.5665	0.093*	
C38	0.8959 (3)	0.0539 (2)	0.5930 (3)	0.1036 (11)	
H38	0.8976	−0.0022	0.5657	0.124*	
C39	0.9992 (3)	0.0443 (2)	0.6328 (3)	0.1048 (11)	
H39	1.0704	−0.0188	0.6334	0.126*	
C40	0.9983 (3)	0.1272 (3)	0.6717 (2)	0.0936 (9)	
H40	1.0689	0.1204	0.6987	0.112*	
C41	0.8928 (2)	0.2212 (2)	0.67094 (18)	0.0717 (6)	
H41	0.8934	0.2783	0.6964	0.086*	
C42	0.5465 (2)	0.21028 (17)	0.73280 (14)	0.0578 (5)	
C43	0.6285 (2)	0.14702 (17)	0.80398 (13)	0.0555 (4)	
H43	0.6941	0.1725	0.8115	0.067*	
C44	0.6106 (2)	0.05441 (17)	0.85759 (14)	0.0565 (5)	
H44	0.5444	0.0319	0.8470	0.068*	
C45	0.68219 (19)	−0.01640 (16)	0.93118 (13)	0.0534 (4)	
C46	0.6598 (2)	−0.11931 (18)	0.97154 (15)	0.0632 (5)	
H46	0.5982	−0.1395	0.9523	0.076*	
C47	0.7274 (2)	−0.19163 (19)	1.03947 (15)	0.0676 (6)	
H47	0.7122	−0.2603	1.0655	0.081*	
C48	0.8172 (2)	−0.1612 (2)	1.06821 (14)	0.0655 (6)	
C49	0.8405 (2)	−0.0588 (2)	1.03070 (16)	0.0661 (5)	
H49	0.9008	−0.0384	1.0513	0.079*	
C50	0.7730 (2)	0.01233 (17)	0.96251 (14)	0.0599 (5)	
H50	0.7886	0.0809	0.9370	0.072*	

### Atomic displacement parameters (Å^2^)

**Table:**

	*U*^11^	*U*^22^	*U*^33^	*U*^12^	*U*^13^	*U*^23^
Cl1	0.0485 (3)	0.0690 (3)	0.0810 (4)	−0.0169 (2)	0.0158 (2)	−0.0153 (3)
Cl2	0.1064 (5)	0.0972 (5)	0.0794 (4)	−0.0360 (4)	−0.0375 (4)	−0.0041 (4)
O1	0.0567 (8)	0.0792 (10)	0.0693 (9)	−0.0061 (7)	−0.0043 (7)	−0.0323 (8)
N1	0.0507 (8)	0.0490 (8)	0.0522 (8)	−0.0209 (7)	−0.0003 (7)	−0.0091 (7)
C1	0.0447 (9)	0.0438 (9)	0.0456 (9)	−0.0159 (7)	−0.0037 (7)	−0.0088 (7)
C2	0.0532 (10)	0.0413 (9)	0.0542 (10)	−0.0113 (8)	−0.0039 (8)	−0.0065 (8)
C3	0.0474 (10)	0.0469 (10)	0.0610 (11)	−0.0053 (8)	−0.0037 (8)	−0.0122 (8)
C4	0.0434 (9)	0.0515 (10)	0.0537 (10)	−0.0143 (8)	0.0019 (7)	−0.0124 (8)
C5	0.0472 (9)	0.0439 (9)	0.0513 (10)	−0.0137 (7)	0.0007 (7)	−0.0075 (7)
C6	0.0413 (8)	0.0424 (9)	0.0455 (9)	−0.0121 (7)	−0.0030 (7)	−0.0097 (7)
C7	0.0437 (9)	0.0446 (9)	0.0475 (9)	−0.0103 (7)	−0.0028 (7)	−0.0094 (7)
C8	0.0404 (9)	0.0526 (10)	0.0519 (10)	−0.0116 (7)	−0.0016 (7)	−0.0144 (8)
C9	0.0452 (9)	0.0573 (11)	0.0525 (10)	−0.0219 (8)	0.0014 (7)	−0.0142 (8)
C10	0.0540 (12)	0.0765 (14)	0.0781 (15)	−0.0321 (11)	0.0114 (10)	−0.0162 (12)
C11	0.0383 (8)	0.0429 (9)	0.0567 (10)	−0.0065 (7)	−0.0001 (7)	−0.0078 (8)
C12	0.0637 (12)	0.0496 (10)	0.0579 (11)	−0.0110 (9)	0.0003 (9)	−0.0107 (9)
C13	0.0793 (15)	0.0576 (12)	0.0578 (12)	−0.0157 (11)	−0.0012 (10)	0.0000 (10)
C14	0.0659 (13)	0.0482 (11)	0.0749 (14)	−0.0127 (9)	0.0006 (11)	−0.0002 (10)
C15	0.0656 (13)	0.0491 (11)	0.0833 (16)	−0.0167 (9)	0.0014 (11)	−0.0177 (10)
C16	0.0586 (11)	0.0534 (11)	0.0616 (12)	−0.0156 (9)	−0.0024 (9)	−0.0131 (9)
C17	0.0436 (9)	0.0602 (11)	0.0550 (11)	−0.0124 (8)	0.0025 (8)	−0.0146 (9)
C18	0.0435 (10)	0.0694 (13)	0.0672 (13)	−0.0024 (9)	−0.0036 (9)	−0.0237 (10)
C19	0.0473 (10)	0.0616 (12)	0.0645 (12)	−0.0061 (9)	−0.0003 (9)	−0.0182 (10)
C20	0.0565 (11)	0.0612 (12)	0.0596 (12)	−0.0132 (9)	−0.0025 (9)	−0.0176 (10)
C21	0.0698 (13)	0.0630 (13)	0.0652 (13)	−0.0134 (10)	0.0016 (10)	−0.0238 (11)
C22	0.0950 (17)	0.0696 (14)	0.0553 (12)	−0.0279 (13)	−0.0025 (11)	−0.0198 (11)
C23	0.0756 (14)	0.0624 (13)	0.0633 (13)	−0.0268 (11)	−0.0144 (11)	−0.0079 (10)
C24	0.0577 (13)	0.0975 (19)	0.0731 (15)	−0.0140 (12)	−0.0089 (11)	−0.0251 (14)
C25	0.0580 (12)	0.1000 (19)	0.0616 (13)	−0.0094 (12)	−0.0046 (10)	−0.0290 (13)
Cl3	0.0766 (4)	0.1135 (5)	0.0664 (3)	−0.0543 (4)	0.0053 (3)	0.0031 (3)
Cl4	0.0807 (4)	0.1019 (5)	0.0778 (4)	−0.0143 (4)	−0.0140 (3)	0.0241 (4)
O2	0.1015 (13)	0.0784 (11)	0.1015 (14)	−0.0568 (11)	−0.0422 (11)	0.0203 (10)
N2	0.0514 (8)	0.0475 (8)	0.0523 (8)	−0.0102 (7)	−0.0031 (7)	−0.0133 (7)
C26	0.0530 (10)	0.0436 (9)	0.0450 (9)	−0.0144 (7)	−0.0042 (7)	−0.0094 (7)
C27	0.0722 (13)	0.0430 (10)	0.0570 (11)	−0.0127 (9)	−0.0058 (9)	−0.0063 (8)
C28	0.0881 (15)	0.0506 (11)	0.0527 (11)	−0.0293 (11)	−0.0055 (10)	0.0007 (9)
C29	0.0638 (12)	0.0673 (12)	0.0469 (10)	−0.0331 (10)	−0.0024 (8)	−0.0049 (9)
C30	0.0489 (10)	0.0575 (11)	0.0525 (10)	−0.0187 (8)	−0.0034 (8)	−0.0090 (8)
C31	0.0475 (9)	0.0437 (9)	0.0455 (9)	−0.0164 (7)	−0.0043 (7)	−0.0080 (7)
C32	0.0471 (9)	0.0430 (9)	0.0516 (9)	−0.0144 (7)	−0.0070 (7)	−0.0068 (7)
C33	0.0512 (10)	0.0461 (9)	0.0491 (9)	−0.0185 (8)	−0.0069 (7)	−0.0039 (7)
C34	0.0487 (9)	0.0544 (10)	0.0476 (9)	−0.0170 (8)	−0.0034 (7)	−0.0110 (8)
C35	0.0556 (12)	0.0802 (15)	0.0641 (13)	−0.0249 (11)	0.0053 (10)	−0.0167 (11)
C36	0.0510 (10)	0.0442 (9)	0.0626 (11)	−0.0136 (8)	−0.0022 (8)	−0.0049 (8)
C37	0.0637 (13)	0.0593 (13)	0.114 (2)	−0.0181 (11)	−0.0014 (13)	−0.0297 (14)
C38	0.0825 (19)	0.0600 (15)	0.170 (3)	−0.0183 (14)	0.015 (2)	−0.0479 (19)
C39	0.0656 (17)	0.0584 (15)	0.164 (3)	0.0017 (12)	0.0044 (18)	−0.0139 (18)
C40	0.0580 (14)	0.0813 (18)	0.121 (3)	0.0008 (12)	−0.0186 (15)	−0.0100 (17)
C41	0.0559 (12)	0.0682 (14)	0.0839 (16)	−0.0075 (10)	−0.0137 (11)	−0.0146 (12)
C42	0.0596 (11)	0.0514 (10)	0.0620 (12)	−0.0247 (9)	−0.0069 (9)	−0.0008 (9)
C43	0.0590 (11)	0.0495 (10)	0.0567 (11)	−0.0203 (9)	−0.0040 (9)	−0.0048 (8)
C44	0.0571 (11)	0.0497 (10)	0.0600 (11)	−0.0191 (8)	−0.0023 (9)	−0.0055 (9)
C45	0.0554 (10)	0.0450 (9)	0.0525 (10)	−0.0126 (8)	0.0050 (8)	−0.0074 (8)
C46	0.0676 (13)	0.0562 (11)	0.0635 (12)	−0.0262 (10)	0.0025 (10)	−0.0044 (10)
C47	0.0739 (14)	0.0528 (11)	0.0627 (13)	−0.0189 (10)	0.0073 (10)	0.0020 (10)
C48	0.0588 (12)	0.0623 (12)	0.0542 (11)	−0.0054 (10)	0.0054 (9)	0.0002 (9)
C49	0.0602 (12)	0.0679 (13)	0.0653 (13)	−0.0170 (10)	−0.0036 (10)	−0.0104 (11)
C50	0.0640 (12)	0.0478 (10)	0.0628 (12)	−0.0167 (9)	−0.0024 (9)	−0.0055 (9)

### Geometric parameters (Å, °)

**Table:**

Cl1—C4	1.7382 (19)	Cl3—C29	1.744 (2)
Cl2—C23	1.742 (2)	Cl4—C48	1.739 (2)
O1—C17	1.217 (2)	O2—C42	1.218 (3)
N1—C9	1.316 (3)	N2—C34	1.319 (3)
N1—C1	1.365 (2)	N2—C26	1.363 (3)
C1—C2	1.410 (3)	C26—C27	1.413 (3)
C1—C6	1.416 (2)	C26—C31	1.414 (3)
C2—C3	1.368 (3)	C27—C28	1.364 (3)
C2—H2	0.9300	C27—H27	0.9300
C3—C4	1.401 (3)	C28—C29	1.397 (3)
C3—H3	0.9300	C28—H28	0.9300
C4—C5	1.362 (3)	C29—C30	1.358 (3)
C5—C6	1.413 (2)	C30—C31	1.412 (3)
C5—H5	0.9300	C30—H30	0.9300
C6—C7	1.431 (2)	C31—C32	1.426 (2)
C7—C8	1.372 (3)	C32—C33	1.374 (3)
C7—C11	1.492 (3)	C32—C36	1.492 (3)
C8—C9	1.428 (3)	C33—C34	1.431 (3)
C8—C17	1.517 (3)	C33—C42	1.506 (3)
C9—C10	1.503 (3)	C34—C35	1.501 (3)
C10—H10A	0.9600	C35—H35A	0.9600
C10—H10B	0.9600	C35—H35B	0.9600
C10—H10C	0.9600	C35—H35C	0.9600
C11—C12	1.381 (3)	C36—C37	1.379 (3)
C11—C16	1.387 (3)	C36—C41	1.390 (3)
C12—C13	1.388 (3)	C37—C38	1.390 (4)
C12—H12	0.9300	C37—H37	0.9300
C13—C14	1.374 (3)	C38—C39	1.364 (5)
C13—H13	0.9300	C38—H38	0.9300
C14—C15	1.365 (4)	C39—C40	1.364 (5)
C14—H14	0.9300	C39—H39	0.9300
C15—C16	1.388 (3)	C40—C41	1.382 (3)
C15—H15	0.9300	C40—H40	0.9300
C16—H16	0.9300	C41—H41	0.9300
C17—C18	1.464 (3)	C42—C43	1.474 (3)
C18—C19	1.319 (3)	C43—C44	1.328 (3)
C18—H18	0.9300	C43—H43	0.9300
C19—C20	1.465 (3)	C44—C45	1.461 (3)
C19—H19	0.9300	C44—H44	0.9300
C20—C21	1.386 (3)	C45—C50	1.389 (3)
C20—C25	1.394 (3)	C45—C46	1.396 (3)
C21—C22	1.384 (4)	C46—C47	1.380 (3)
C21—H21	0.9300	C46—H46	0.9300
C22—C23	1.375 (4)	C47—C48	1.369 (4)
C22—H22	0.9300	C47—H47	0.9300
C23—C24	1.371 (4)	C48—C49	1.387 (3)
C24—C25	1.377 (3)	C49—C50	1.378 (3)
C24—H24	0.9300	C49—H49	0.9300
C25—H25	0.9300	C50—H50	0.9300
			
C9—N1—C1	118.46 (16)	C34—N2—C26	118.56 (16)
N1—C1—C2	118.14 (16)	N2—C26—C27	118.09 (17)
N1—C1—C6	122.92 (16)	N2—C26—C31	123.31 (17)
C2—C1—C6	118.93 (16)	C27—C26—C31	118.60 (18)
C3—C2—C1	121.07 (17)	C28—C27—C26	121.1 (2)
C3—C2—H2	119.5	C28—C27—H27	119.4
C1—C2—H2	119.5	C26—C27—H27	119.4
C2—C3—C4	119.13 (17)	C27—C28—C29	119.23 (19)
C2—C3—H3	120.4	C27—C28—H28	120.4
C4—C3—H3	120.4	C29—C28—H28	120.4
C5—C4—C3	122.07 (17)	C30—C29—C28	122.08 (19)
C5—C4—Cl1	119.37 (15)	C30—C29—Cl3	119.69 (17)
C3—C4—Cl1	118.55 (14)	C28—C29—Cl3	118.22 (16)
C4—C5—C6	119.44 (17)	C29—C30—C31	119.53 (19)
C4—C5—H5	120.3	C29—C30—H30	120.2
C6—C5—H5	120.3	C31—C30—H30	120.2
C5—C6—C1	119.35 (16)	C30—C31—C26	119.43 (17)
C5—C6—C7	122.85 (16)	C30—C31—C32	123.25 (17)
C1—C6—C7	117.78 (15)	C26—C31—C32	117.30 (16)
C8—C7—C6	118.00 (16)	C33—C32—C31	118.61 (16)
C8—C7—C11	122.04 (16)	C33—C32—C36	121.53 (16)
C6—C7—C11	119.96 (15)	C31—C32—C36	119.79 (16)
C7—C8—C9	120.17 (16)	C32—C33—C34	119.91 (17)
C7—C8—C17	120.18 (17)	C32—C33—C42	121.42 (17)
C9—C8—C17	119.57 (16)	C34—C33—C42	118.64 (17)
N1—C9—C8	122.56 (16)	N2—C34—C33	122.17 (17)
N1—C9—C10	116.73 (18)	N2—C34—C35	116.12 (18)
C8—C9—C10	120.69 (18)	C33—C34—C35	121.69 (18)
C9—C10—H10A	109.5	C34—C35—H35A	109.5
C9—C10—H10B	109.5	C34—C35—H35B	109.5
H10A—C10—H10B	109.5	H35A—C35—H35B	109.5
C9—C10—H10C	109.5	C34—C35—H35C	109.5
H10A—C10—H10C	109.5	H35A—C35—H35C	109.5
H10B—C10—H10C	109.5	H35B—C35—H35C	109.5
C12—C11—C16	119.32 (19)	C37—C36—C41	118.8 (2)
C12—C11—C7	119.69 (17)	C37—C36—C32	120.9 (2)
C16—C11—C7	120.99 (18)	C41—C36—C32	120.26 (19)
C11—C12—C13	120.0 (2)	C36—C37—C38	120.0 (3)
C11—C12—H12	120.0	C36—C37—H37	120.0
C13—C12—H12	120.0	C38—C37—H37	120.0
C14—C13—C12	120.2 (2)	C39—C38—C37	120.4 (3)
C14—C13—H13	119.9	C39—C38—H38	119.8
C12—C13—H13	119.9	C37—C38—H38	119.8
C15—C14—C13	120.3 (2)	C40—C39—C38	120.2 (3)
C15—C14—H14	119.9	C40—C39—H39	119.9
C13—C14—H14	119.9	C38—C39—H39	119.9
C14—C15—C16	120.1 (2)	C39—C40—C41	120.1 (3)
C14—C15—H15	120.0	C39—C40—H40	119.9
C16—C15—H15	120.0	C41—C40—H40	119.9
C11—C16—C15	120.2 (2)	C40—C41—C36	120.4 (3)
C11—C16—H16	119.9	C40—C41—H41	119.8
C15—C16—H16	119.9	C36—C41—H41	119.8
O1—C17—C18	120.89 (18)	O2—C42—C43	122.00 (19)
O1—C17—C8	119.33 (18)	O2—C42—C33	119.04 (19)
C18—C17—C8	119.78 (17)	C43—C42—C33	118.92 (17)
C19—C18—C17	125.35 (19)	C44—C43—C42	121.33 (19)
C19—C18—H18	117.3	C44—C43—H43	119.3
C17—C18—H18	117.3	C42—C43—H43	119.3
C18—C19—C20	126.18 (19)	C43—C44—C45	127.8 (2)
C18—C19—H19	116.9	C43—C44—H44	116.1
C20—C19—H19	116.9	C45—C44—H44	116.1
C21—C20—C25	117.8 (2)	C50—C45—C46	118.0 (2)
C21—C20—C19	120.6 (2)	C50—C45—C44	123.49 (18)
C25—C20—C19	121.6 (2)	C46—C45—C44	118.5 (2)
C22—C21—C20	121.2 (2)	C47—C46—C45	121.2 (2)
C22—C21—H21	119.4	C47—C46—H46	119.4
C20—C21—H21	119.4	C45—C46—H46	119.4
C23—C22—C21	119.1 (2)	C48—C47—C46	119.3 (2)
C23—C22—H22	120.5	C48—C47—H47	120.4
C21—C22—H22	120.5	C46—C47—H47	120.4
C24—C23—C22	121.2 (2)	C47—C48—C49	121.2 (2)
C24—C23—Cl2	118.6 (2)	C47—C48—Cl4	119.60 (18)
C22—C23—Cl2	120.08 (19)	C49—C48—Cl4	119.2 (2)
C23—C24—C25	119.1 (2)	C50—C49—C48	119.0 (2)
C23—C24—H24	120.4	C50—C49—H49	120.5
C25—C24—H24	120.4	C48—C49—H49	120.5
C24—C25—C20	121.4 (2)	C49—C50—C45	121.3 (2)
C24—C25—H25	119.3	C49—C50—H50	119.4
C20—C25—H25	119.3	C45—C50—H50	119.4
			
C9—N1—C1—C2	−176.90 (17)	C34—N2—C26—C27	176.12 (18)
C9—N1—C1—C6	2.7 (3)	C34—N2—C26—C31	−3.2 (3)
N1—C1—C2—C3	−179.97 (18)	N2—C26—C27—C28	−179.62 (19)
C6—C1—C2—C3	0.4 (3)	C31—C26—C27—C28	−0.2 (3)
C1—C2—C3—C4	0.5 (3)	C26—C27—C28—C29	−0.9 (3)
C2—C3—C4—C5	−1.0 (3)	C27—C28—C29—C30	1.3 (3)
C2—C3—C4—Cl1	−179.51 (16)	C27—C28—C29—Cl3	−179.09 (17)
C3—C4—C5—C6	0.5 (3)	C28—C29—C30—C31	−0.7 (3)
Cl1—C4—C5—C6	179.04 (14)	Cl3—C29—C30—C31	179.77 (15)
C4—C5—C6—C1	0.4 (3)	C29—C30—C31—C26	−0.5 (3)
C4—C5—C6—C7	−177.66 (17)	C29—C30—C31—C32	177.63 (18)
N1—C1—C6—C5	179.54 (17)	N2—C26—C31—C30	−179.74 (17)
C2—C1—C6—C5	−0.9 (3)	C27—C26—C31—C30	0.9 (3)
N1—C1—C6—C7	−2.3 (3)	N2—C26—C31—C32	2.0 (3)
C2—C1—C6—C7	177.31 (16)	C27—C26—C31—C32	−177.30 (17)
C5—C6—C7—C8	177.51 (17)	C30—C31—C32—C33	−176.60 (17)
C1—C6—C7—C8	−0.6 (3)	C26—C31—C32—C33	1.5 (3)
C5—C6—C7—C11	−2.4 (3)	C30—C31—C32—C36	0.4 (3)
C1—C6—C7—C11	179.46 (16)	C26—C31—C32—C36	178.50 (17)
C6—C7—C8—C9	2.9 (3)	C31—C32—C33—C34	−3.8 (3)
C11—C7—C8—C9	−177.17 (17)	C36—C32—C33—C34	179.30 (17)
C6—C7—C8—C17	−173.98 (16)	C31—C32—C33—C42	174.06 (17)
C11—C7—C8—C17	6.0 (3)	C36—C32—C33—C42	−2.8 (3)
C1—N1—C9—C8	−0.2 (3)	C26—N2—C34—C33	0.8 (3)
C1—N1—C9—C10	−178.50 (17)	C26—N2—C34—C35	179.19 (17)
C7—C8—C9—N1	−2.6 (3)	C32—C33—C34—N2	2.7 (3)
C17—C8—C9—N1	174.28 (18)	C42—C33—C34—N2	−175.19 (18)
C7—C8—C9—C10	175.59 (19)	C32—C33—C34—C35	−175.55 (18)
C17—C8—C9—C10	−7.5 (3)	C42—C33—C34—C35	6.5 (3)
C8—C7—C11—C12	111.0 (2)	C33—C32—C36—C37	69.7 (3)
C6—C7—C11—C12	−69.0 (2)	C31—C32—C36—C37	−107.1 (2)
C8—C7—C11—C16	−69.5 (3)	C33—C32—C36—C41	−110.8 (2)
C6—C7—C11—C16	110.4 (2)	C31—C32—C36—C41	72.3 (3)
C16—C11—C12—C13	0.0 (3)	C41—C36—C37—C38	0.6 (4)
C7—C11—C12—C13	179.45 (19)	C32—C36—C37—C38	−179.9 (3)
C11—C12—C13—C14	−0.7 (4)	C36—C37—C38—C39	0.9 (5)
C12—C13—C14—C15	1.0 (4)	C37—C38—C39—C40	−1.2 (6)
C13—C14—C15—C16	−0.5 (4)	C38—C39—C40—C41	0.0 (6)
C12—C11—C16—C15	0.5 (3)	C39—C40—C41—C36	1.5 (5)
C7—C11—C16—C15	−178.98 (18)	C37—C36—C41—C40	−1.8 (4)
C14—C15—C16—C11	−0.2 (3)	C32—C36—C41—C40	178.7 (2)
C7—C8—C17—O1	91.2 (2)	C32—C33—C42—O2	−119.1 (3)
C9—C8—C17—O1	−85.7 (2)	C34—C33—C42—O2	58.8 (3)
C7—C8—C17—C18	−88.1 (2)	C32—C33—C42—C43	63.1 (3)
C9—C8—C17—C18	95.0 (2)	C34—C33—C42—C43	−119.0 (2)
O1—C17—C18—C19	−174.0 (2)	O2—C42—C43—C44	−5.8 (4)
C8—C17—C18—C19	5.3 (4)	C33—C42—C43—C44	171.9 (2)
C17—C18—C19—C20	176.6 (2)	C42—C43—C44—C45	−179.3 (2)
C18—C19—C20—C21	−159.6 (2)	C43—C44—C45—C50	8.2 (3)
C18—C19—C20—C25	19.1 (4)	C43—C44—C45—C46	−170.8 (2)
C25—C20—C21—C22	−3.5 (4)	C50—C45—C46—C47	−1.2 (3)
C19—C20—C21—C22	175.3 (2)	C44—C45—C46—C47	177.8 (2)
C20—C21—C22—C23	0.6 (4)	C45—C46—C47—C48	0.6 (3)
C21—C22—C23—C24	2.7 (4)	C46—C47—C48—C49	0.5 (3)
C21—C22—C23—Cl2	−175.03 (19)	C46—C47—C48—Cl4	−179.29 (17)
C22—C23—C24—C25	−2.9 (4)	C47—C48—C49—C50	−1.0 (3)
Cl2—C23—C24—C25	174.8 (2)	Cl4—C48—C49—C50	178.82 (17)
C23—C24—C25—C20	−0.2 (5)	C48—C49—C50—C45	0.3 (3)
C21—C20—C25—C24	3.3 (4)	C46—C45—C50—C49	0.8 (3)
C19—C20—C25—C24	−175.5 (3)	C44—C45—C50—C49	−178.2 (2)

### Hydrogen-bond geometry (Å, °)

**Table:**

Cg1 is the centroid of the C20–C25 ring.

**Table:**

*D*—H···*A*	*D*—H	H···*A*	*D*···*A*	*D*—H···*A*
C38—H38···Cg1^i^	0.93	2.93	3.458 (3)	117

Symmetry codes: (i) −*x*+2, −*y*, −*z*+1.
